# Late-Maturity Alpha-Amylase in Wheat (*Triticum aestivum*) and Its Impact on Fresh White Sauce Qualities

**DOI:** 10.3390/foods10020201

**Published:** 2021-01-20

**Authors:** Galex K. S. Neoh, Mark J. Dieters, Keyu Tao, Glen P. Fox, Phuong T. M. Nguyen, Robert G. Gilbert

**Affiliations:** 1 Joint International Research Laboratory of Agriculture and Agri-Product Safety, College of Agriculture, Yangzhou University, Yangzhou 225009, China; k.neoh@uq.edu.au (G.K.S.N.); k.tao@uqconnect.edu.au (K.T.); 2 Centre for Nutrition and Food Science, Queensland Alliance for Agriculture and Food Innovation (QAAFI), The University of Queensland, Brisbane, QLD 4072, Australia; m.dieters@uq.edu.au (M.J.D.); gpfox@ucdavis.edu (G.P.F.); 3 School of Agriculture and Food Sciences, The University of Queensland, Brisbane, QLD 4072, Australia; p.nguyen2@uq.edu.au; 4 Department of Food Science and Technology, University of California Davis, Davis, CA 95616, USA

**Keywords:** late-maturity alpha-amylase, white sauce, starch structure, gelatinization, gelation, texture analyzer

## Abstract

When wheat experiences a cold-temperature ‘shock’ during the late stage of grain filling, it triggers the abnormal synthesis of late-maturity *α*-amylase (LMA). This increases the enzyme content in affected grain, which can lead to a drastic reduction in falling number (FN). By commercial standards, a low FN is taken as an indication of inferior quality, deemed unsuitable for end-product usage. Hence, LMA-affected grains are either rejected or downgraded to feed grade at the grain receiving point. However, previous studies have found no substantial correlation between low FN-LMA and bread quality. The present study extends previous investigations to semi-solid food, evaluating the physical quality of fresh white sauce processed from LMA-affected flour. Results show that high-LMA flours had low FNs and exhibited poor pasting characteristics. However, gelation occurred in the presence of other components during fresh white sauce processing. This demonstrates that LMA-affected flours may have new applications in low-viscosity products.

## 1. Introduction

Late-maturity *α*-amylase (LMA) is a weather-induced field incident in wheat (*Triticum aestivum*), and is a consequence of unfavourable weather during the growing season. LMA is primarily triggered by a sudden drop in growing temperature during the post-anthesis stage, but can also be triggered by a sudden increase in growing temperature [1,2]. This change in weather leads to the recurrence of *α*-amylase synthesis in grains, which is not normal for plant development during this stage. Interestingly, LMA does not have any impact on grain morphology or composition [3,4].

LMA is a major concern to the wheat industry, as affected grains contain a larger amount than normal of high-pI *α*-amylase, which can lower the falling number (FN) to near or below commercial standards. The FN test is a standardized method (AACC 56-81.04) employed globally to assess grain quality at receival points. The testing process involves heating flour slurry in a viscometric tube at boiling temperature. A standard plunger is then released from the top of the viscometric tube and FN is the time taken in seconds for the plunger to fall through the flour slurry. A low FN indicates that the flour slurry has low viscosity, that is attributed to a high amount of *α*-amylase in the flour. The wheat industry views low FN as an indication of poor-quality grains that carry a considerable amount of risk, which could potentially have an adverse impact on processing and end-product qualities. In most countries, 300 s is the standard of reference to pass the FN test. The threshold value for the FN test can range between 250 and 450 s, depending on the specific market requirements of the country [5]. Flour milled from an LMA-affected grain can have FN below 100 s, but the FN generally ranges from 150 s to slightly below the FN threshold value [3]. Such flours are sold at a discounted rate or downgraded to feed grade [6]. Low FN has costed the grains industries in the Pacific Northwest of the US more than USD 140 million in 2016 [5]. An economic assessment by Kingwell and Carter [6] in 2014 reported that Western Australia would be likely to suffer an AUD 18 million loss annually, based on the current industry standards.

Unlike pre-harvest sprouting (PHS), another weather-induced field incident in wheat which has well-documented evidence of its detrimental effects on end-product quality, no study to date has reported any correlation between a low FN associated with LMA and poor end-product quality. Ral et al. [7] evaluated the wheat-baking quality of LMA flour and found it had lower pasting viscosity, endothermic transition temperature and FN than non-affected flour. However, the bread processed from LMA flour had greater loaf volume and its crust had a better visual appeal, as characterized by a darker golden hue. These findings suggest that the higher level of *α*-amylase in LMA-affected flour contributes to increased starch hydrolysis, which eventually leads to an increased amount of fermentable sugars for the yeast to metabolize, which in turn leads to an increased loaf volume. A subsequent study by Newberry et al. [8] assessed seven quality parameters of bread made from LMA-affected flours. It was shown that there was no strong correlation between low FN as a consequence of LMA, and bread quality. These studies demonstrated that LMA did not have any adverse effects on end-product quality and questioned the relevance of low FN as an indication of grain quality relating to the case of LMA.

The assumption that LMA-affected grains are of poorer quality is solely based on low FN and the success with the FN test for accurately predicting the quality of PHS-affected grains. Studies have already shown evidence that LMA has negligible impact on bread quality [7,8]. Nevertheless, there is still lack of information on LMA impact on other food matrices, such as semi-solid and liquid food (e.g., dressings, sauces and purées). As the food matrices of solid, semi-solid and liquid food are vastly different; LMA impact on semi-solid food quality could potentially be different to that of solid foods. Wheat flour and starch are commonly used as thickeners and gelling agents in a wide array of semi-liquid/solid food products such as sauces, gravies and dressings. Its thickening characteristics are related to the starch granule morphology and *α*-amylase activity [9]. Previous studies reported no differences in the grain compositions and starch structure between non-LMA- and LMA-affected wheat [7,10]. However, the excessive amount of *α*-amylase in LMA affected grains drastically reduced flour gelation ability due to starch liquefaction and have very low final viscosities, although it seems to have no detrimental impact on bread quality. The aim of the present study was to elucidate LMA impact on semi-solid end-product physical qualities by assessing the textural properties in a product processed from LMA-affected flours. Two different LMA-susceptible cultivars were chosen to examine the influence of LMA on the end-product from these cultivars. In addition, the LMA contents in whole-wheat and white flour were also evaluated to determine its amount in different grain fractions.

For this study, fresh white sauce was chosen as the semi-solid food of interest for evaluation, due to its simple food components (oil, flour and milk) and their wide range of uses as a base for other sauces. The three objectives of this study are as follows: (1) determine LMA content in whole-wheat flour (WWF) and white flour (WF), (2) evaluate the pasting properties of WWF and WF milled from LMA-affected grains and (3) assess the impact of LMA on the textural properties of freshly prepared white sauce. The study used a commercial white sauce purchased from a local major supermarket as reference.

These objectives should broaden current knowledge of LMA, verifying earlier findings and provide new insights into the actual impact of LMA on different types of food product. Furthermore, the findings could also be useful information for the wheat industry for managing LMA-affected grains.

## 2. Materials and Methods

### 2.1. Materials

Skimmed milk, vegetable oil, table salt and commercial white sauce were purchased from a major supermarket chain in Brisbane, Australia. Xanthan gum was from the Melbourne Food Ingredient Depot (Melbourne, Australia). Antibodies and colour developer (3,3′,5,5′-tetramethylbenzidine substrate) for LMA content determination were from South Australia Research and Development Institute (Hartley Grove, SA, Australia) and ELISA Systems (Windsor, QLD, Australia), respectively. Unless stated otherwise, all chemicals were from Sigma-Aldrich (Castle Hill, NSW, Australia) and used as received.

### 2.2. Plant Rearing and LMA Induction

Based on the current LMA status as classified by the Australian Grains Research and Development Corporation (GRDC) and University of Adelaide (UA), two local Australian wheat (*Triticum aestivum L.*) cultivars, Chara (high-LMA) and SEA Condamine (intermediate-LMA), were chosen for the evaluation of processing and end-product quality. The plants were reared in a climate-controlled glasshouse under natural light supplemented with high intensity LED lights from November to March 2020, at the University of Queensland, Brisbane, Australia, as described in Mrva and Mares [11], with minor modifications. Nine sound grains were sown and spaced equally in a 125-mm ANOVA pot. For each cultivar, there were four biological replicates per treatment (control/LMA induction), and each replicate was a plot consisting of 24 pots. The pots were arranged in a randomized split-plot design, with each replicate allocated to one of the four benches in the glasshouse. The pots were hand-watered daily as required. The temperature was programmed on a diurnal cycle of 25 °C and 15 °C during the day and night, respectively, on 12/12 h day/night cycle. On 28 days post-anthesis (DPA), secondary tillers were removed and pots due for LMA induction were relocated to a 12 °C cold room chamber set to 12/12 h day/night cycle, for 10 days to simulate a cold temperature ‘shock’. Subsequently, the cold-treated pots were transferred back to the glasshouse until maturity. Control pots remained in the glasshouse throughout the rearing period. Upon harvest ripeness, the spikes were threshed using a Wintersteiger LD 180 thresher (Winterseiger Co., Ried, Austria). Grains from each biological replicate plot of the same treatment were mixed and half of the pooled grains were milled as whole-wheat flour and the other half as white flour. Whole grains were milled on a Perten Lab Mill 3310 fitted with type 2 (fine) grinding discs (PerkinElmer Co., Hägersten, Sweden) and Brabender Quadrumat^®^ Junior (Brabender GmbH & CO. KG., Duisburg, Germany), fitted with a standard 240 μm sifter, to obtain the whole-wheat and white flour respectively. Following milling, whole-wheat flour was passed through a 0.55 mm sieve. Approximately 100 g of whole-wheat and white flour per biological replicate of the same treatment were used for subsequent analysis.

### 2.3. Total Enzymatic Activities and LMA Content Determination

The total *α*-amylase activity was determined using the Ceralpha Method (Megazyme International Ireland Ltd., Bray, Ireland) in 96-well microplate format. All chemical reagents used were provided in the assay kit. 50 mg of a sample was extracted with 600 μL extraction buffer at 40 °C in a 1.5 mL microcentrifuge tube and left for 20 min. Subsequently, the samples were centrifuged at 3000× *g* for 10 min, and 150 µL of the supernatant was aliquoted into the wells of a microtiter plate. Thereafter, 20 μL of the Amylase HR Reagent (substrate) was added into each well using a multichannel pipette at 30 s intervals per column. The mixtures were incubated at room temperature for 40 min. At the end of the incubation period, 300 μL of stopping reagent was added to the wells in the same sequence and time intervals. The absorbance was read at 400 nm and its total *α*-amylase activity expressed as Ceralpha-units (CU)/g of sample, CU being defined as the amount of enzyme required in the presence of excess thermostable *α*-glucosidase to release 1 mmol of *p*-nitrophenol from the substrate at room temperature in 1 min. The LMA content in 50 mg of WF and WWF were determined using an LMA-specific sandwich enzyme-linked immunosorbent assay (ELISA), which binds exclusively to the high pI isoform of wheat *α*-amylase that is identical to LMA, as described in Barrero et al. [12]. LMA content was expressed as its absorbance (OD) value. All spectrophotometric measurements were performed using a FLUORstar OPTIMA microplate reader (BMG LABTECH Co., Ortenberg, Germany). Samples were randomly allocated to the 96-well microtiter plate according to a row-column design generated by DeltaGen version 0.03 for both enzymatic assays [13].

### 2.4. Stirring Number (SN) Determination

Stirring number was determined using a Rapid Visco Analyser (Newport Scientific, Warriewood, NSW, Australia), in accordance with the AACC International Method 22-08.02. A sample of 4 g WWF and 3.5 g WF (dry basis), respectively, was used for the measurements. The flour was added into a canister containing 25 mL distilled water and was gently dispersed by hand using the Rapid Visco Analyser (RVA) paddle. Shortly after, the canister was loaded onto the RVA to initiate the test. The temperature was held at 95 °C. The sample was stirred at 960 rpm for the first 10 s, which was subsequently reduced to 160 rpm for the remaining 170 s of the test. The FN was calculated from the SN values, as defined by its apparent viscosity in rapid visco units (RVU), based on Tordenmalm et al. [14].

### 2.5. Pasting Properties

The flour pasting characteristics were assessed using a Rapid Visco Analyser (Newport Scientific, Warriewood, NSW, Australia) in accordance with AACC International Method 76-21.02, standard RVA profile (RVA STD 1), measuring pasting temperature, peak viscosity, holding viscosity, final (peak) viscosity, breakdown and total setback. Samples weighing 3.5 g of each of WWF and WF (dry basis), respectively, were used for the analysis. The sample preparation procedures were the same as in the FN test described in the preceding section. The flour slurry in the canister was first held at 50 °C for 1 min, before heating to 95 °C in 3.7 min, then held at 95 °C for 1.5 min, followed by cooling to 50 °C in 3.7 min, and lastly held at 50 °C for 2 min, at which time the test was concluded. At the start of the test, the rotor speed was 960 rpm for 10 s, and then reduced to 160 rpm for the remaining time. The RVA parameter values, which include peak viscosity (PV), peak time (PT), trough viscosity (TV) and final viscosity (FV), were calculated with the Thermocline software provided with the instrument. Viscosity was expressed in RVU and time in s.

### 2.6. Fresh White Sauce Preparation and Gelation Properties

A Rapid Visco Analyser (Newport Scientific, Warriewood, NSW, Australia) was employed to prepare the white sauce, using a formulation based on that given by Arocas et al. [15] with minor modifications. The white sauce consisted of skimmed milk (91.25%, *w*/*w*), white flour (6% *w*/*w*), vegetable oil (2.5% *w*/*w*) and table salt (0.25% *w*/*w*). The skimmed milk for the white sauce mixture containing xanthan (0.15% *w*/*w*) was reduced to 91.1% (*w*/*w*). The *w*/*w* ratio of all other ingredients used was the same as that without xanthan. The pre-weighed ingredients were transferred to the RVA canister prior to making the white sauce. The canister was loaded onto the RVA and was heated from 50 to 95 °C in 4 min, held at 95 °C for 3 min, cooled to 70 °C in 4 min and maintained at 70 °C for 3 min. The mixture was stirred at 960 rpm for 10 s, and then the speed was reduced and maintained at 200 rpm for the remaining time. The white sauce was maintained at 70 °C using a water bath for 2 min to settle down the white sauce emulsion before performing subsequent analyses. Commercial white sauce powder (Gravox White, Seven Hills, NSW, Australia) was mixed with skimmed milk in proportion (*w*/*w*) as per the manufacturer’s instructions to acquire a total of 25 g, and was prepared by the same procedures as detailed above.

### 2.7. Fresh White Sauce Texture Properties

A TA XT.PLUS texture analyser (Stable Micro System Ltd., Surrey, UK) equipped with a 10 kg load cell was used to perform a back extrusion technique to evaluate white sauce physical qualities. The equipment and test set-up were based on Arocas et al. [16] with modification. A 32 mm-diameter back extrusion cell, which provides a 6 mm annulus gap, was used. The white sauce was subjected to a single compression at a rate of 1 mm/s to a distance of 15 mm. The trigger force was 10 g and the compression depth was approximately 44% of the white sauce height. The four parameters obtained from the back extrusion test were firmness (in g, the gravitational unit), consistency (g s), cohesiveness (g) and work of cohesion (g s). Firmness and cohesiveness are respectively the maximum force required for compressing and extruding the white sauce. Consistency and index of viscosity (IOV) are the positive and negative area under curve of the force-time graph. The Exponent Connect software provided by the instrument manufacturer was used to generate the parameter values.

### 2.8. Statistical Analysis

Unless stated otherwise, all analyses were performed in biological quadruplicates, and values are presented as mean ± standard deviation (SD). Analysis of variance (ANOVA) was performed using GraphPad Prism version 8.4.2 for Windows (GraphPad Software, La Jolla, CA, USA) and SPSS version 16 (SPSS Inc., Chicago, IL, USA), with mean comparison by Tukey’s multiple comparison test and statistical significance at *p* < 0.05.

## 3. Results and Discussion

### 3.1. LMA Content and Total α-Amylase Activity in Whole-Wheat Flour (WWF) and White Flour (WF)

Here and in the following, control and LMA-affected flours refer to flours milled from wheat grown in normal condition and those that underwent addition cold treatment on 28 DPA for 10 days respectively. LMA manifestation in wheat is highly variable and it may not always occur after experiencing a cold temperature ‘shock’ [1]. Hence, this study employs a high pI ELISA assay specific to the LMA isoform for detecting the presence of LMA, and to ensure that the samples used for subsequent analysis were LMA-induced. It is important to note that LMA is defined by specific condition, at which its synthesis is induced by ‘cold-temperature’ during late post-anthesis stage. Hence, LMA detected in control WWF and WF samples by the LMA-ELISA assay is presumably the inherent high pI *α*-amylase, of which its presence is normal to that observed in sound grains [17].

**Figure 1:**
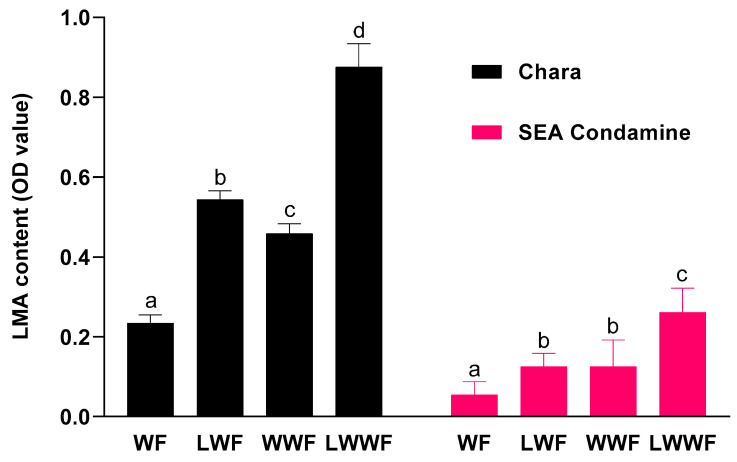
LMA content in white and whole-wheat flour milled from wheat grown under different condition during late post-anthesis stage. (WF) control white flour; (LWF) LMA-affected white flour; (WWF) control whole-wheat flour; (LWWF) LMA-affected whole-wheat flour. Respective cultivars marked by different letters indicate significant differences (*p* ≤ 0.05).

As shown in Figure 1, the increase in high pI *α*-amylase in LMA-affected WWF and WF for Chara and SEA Condamine indicated that LMA was induced with cold-temperature treatment at 28 DPA. The LMA content of Chara flours ranged between 0.23 and 0.87 OD. Their LMA-affected WWF and WF had LMA content significantly higher than those in the corresponding controls. For SEA Condamine, the LMA content ranged between 0.06 and 0.26 OD. Similarly, both the LMA-affected WWF and WF were also significantly higher than the corresponding controls. For both cultivars, the average percentage of non-starchy endosperm fraction in WWF was 18.4%. However, the LMA contents of their control WWFs was ~2.2 times higher than those of the corresponding WFs. This suggests that the amount of high pI *α*-amylase in the non-starchy endosperm fraction is higher in proportion than in an equal weight of flour fraction; other studies have reported similar findings [18,19]. The LMA content increment in LMA-affected WWF and WF were of similar magnitude (~2.1-fold) to those of the corresponding controls for both cultivars. It is uncertain whether this proportionate increment observed was random or determined by specific conditions.

SEA Condamine LMA-affected WF and WWF did not develop the LMA content of an intermediate LMA cultivar, which typically ranged between 0.4 and 0.6 OD values for WWF [20]. Previous studies have demonstrated that the time for inducing LMA by cold-temperature shock was between 25 and 35 DPA; this time was used here in cultivation [11,21]. However, a recent study reported that the optimal time for inducing LMA varies with the rate of grain development and the stage of grain maturity [20]. In addition, wheat that received a cold-temperature shock six days prior or past the optimum time have LMA contents up to five-fold lower. Hence, the optimum time for LMA synthesis may have passed for when SEA Condamine was relocated to the cold room chamber. This suggests that SEA Condamine may have a relatively shorter grain filling period than those of cultivars reported in the previous study [11,21]. It is challenging to determine the optimal time for LMA induction during the cultivation, as these optimal conditions are multifactorial.

**Figure 2:**
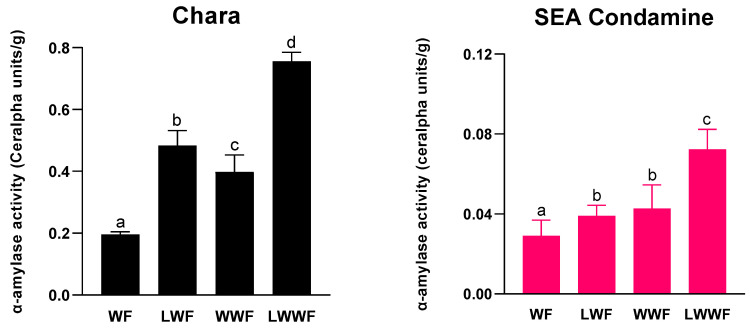
Total *α*-amylase activity of white and whole-wheat flours milled from wheat grown under different condition during late post-anthesis stage. (WF) control white flour; (LWF) LMA-affected white flour; (WWF) control whole-wheat flour; (LWWF) LMA-affected whole-wheat flour. Respective cultivars marked by different letters indicate significant differences (*p* ≤ 0.05).

Total *α*-amylase activity was measured to examine the hydrolytic efficiency of LMA in wheat flour. Shown in Figure 2, the total *α*-amylase activity for Chara ranged between 0.20 and 0.76 CU/g, and for SEA Condamine, between 0.03 and 0.07 CU/g. All LMA-affected flours have total *α*-amylase activity significantly higher than their corresponding controls. Similar to that observed for LMA content, there was only a minor difference in total *α*-amylase activity in SEA Condamine LMA-affected WF and the control WWF.

### 3.2. LMA Impact on Flours FNs

SN tests were performed due to their advantage of needing half the amount of flour sample (3.5–4 g) than that required for the FN test (7 g). Furthermore, results of a joint study which involved 17 collaborators showed that there was a strong linear relationship between FN and SN test (*r* = 0.993) for 36 flour samples with a wide range of FNs that were analysed [14]. As shown in Table 1, Chara flours have FNs between 69 and 375 s. The control WWF and WF FNs were significantly different to their LMA-affected counterpart. The addition of 2 mM silver nitrate to LMA-affected flours restored their FNs and was significantly higher than their corresponding controls. For SEA Condamine, the FNs were between 360 and 389 s. All flours have FNs significantly higher than the minimum commercial requirement by a good margin. Their control and LMA-affected WF have similar FNs, whilst LMA-affected WWF has significantly lower FN than its control. Silver nitrate also restored the FN of LMA-affected WF from 374 to 387 s, slightly higher than its control FN-380 s. On the other hand, LMA-affected WWF FN increased considerably in the presence of silver nitrate, from 360 to 389 s, and had FNs significantly higher than its control FN. Both cultivars WFs have higher FNs than their respective WWFs for wheat that were grown in normal conditions and those that underwent cold treatment. A lower FN represents greater liquefaction of the flour slurry during the experiment. This indicates that there was higher enzymatic activity in WWF during the measurement, which concurred with earlier results (Figure 1). Silver nitrate was also used when performing FN test for Chara and SEA Condamine control flours to examine the impact of inherent *α*-amylase on FN. Results show that inherent *α*-amylase in Chara control WF and WWF reduced their innate FN by 18.4% and 34.1%, respectively (Table 1). For SEA Condamine, inherent *α*-amylase in their control WF and WWF reduced their FN by 1.04% and 5.6%, respectively. These results are consistent to earlier LMA and total *α*-amylase findings, at which high enzyme content and activity were detected in the bran fraction, which contributed to a greater reduction in the FNs of WWF than that of WF.

Unlike the case for PHS, there is no evidence of substantial changes in the starch properties and other non-polysaccharides components of LMA-affected grains [7,10,22]. Thus, any discrepancy in FNs between control and LMA-affected flours is attributed solely to inherent *α*-amylase. Silver nitrate can deactivate enzymatic activity, but at the low concentration used, did not alter the flour-inherent pasting properties [23]. Thus, it was used on LMA-affected flour to inhibit LMA activity, and accordingly examine LMA impact on FNs. For Chara, silver nitrate restored the FNs of LMA-affected WWFs and WFs, which were respectively 50% and 21% higher than their corresponding controls (Table 1). This shows that the inherent *α*-amylase in Chara grown in normal conditions was sufficient to reduce its primary FNs to close or below the standard threshold value of 300 s for commercial FNs. This is a typical characteristic of high LMA cultivars, although LMA expression is irregular and high LMA-prone cultivars may express low amounts of LMA [1,24]. As demonstrated in previous studies, high LMA prone cultivars such as Seri and Kennedy among others express LMA, regardless of growing conditions, although their level of expression can vary greatly [20,24]. Hence, these LMA-prone cultivars may fail the FN test. In contrast to what was observed for Chara, the SEA Condamine LMA-affected flours FNs did not increase as drastically upon being added to silver nitrate, due to a low level of enzyme activity.

### 3.3. Pasting Properties of LMA-Affected Flours

**Figure 3:**
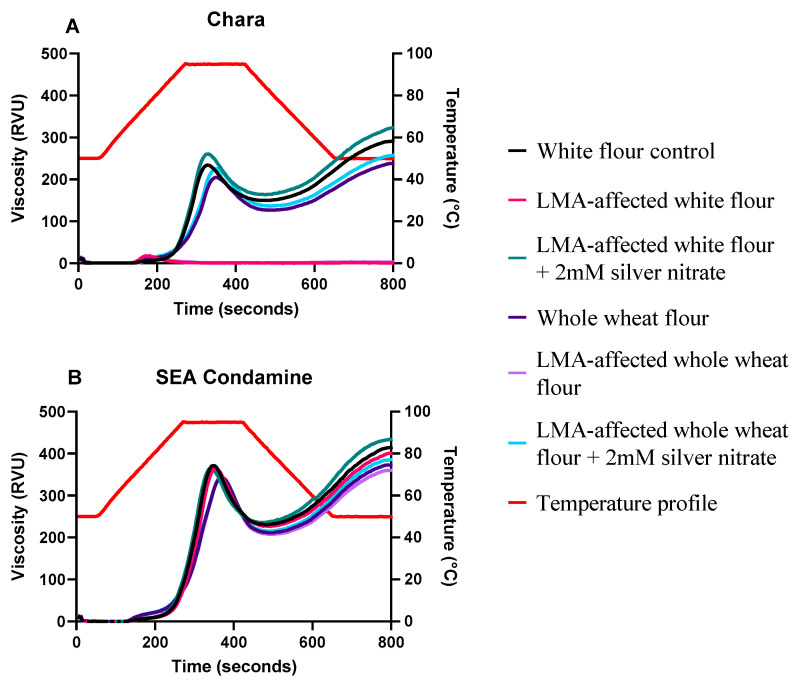
RVA Pasting Profiles of (**A**) Chara and (**B**) SEA Condamine. RVA viscogram illustrating pasting behaviour of individual cultivars control and LMA affected-flours with and without 2 mM silver nitrate.

The pasting profiles of Chara and SEA Condamine flours are given in Figure 3A,B, with RVA parameter values given in Table 2. Other than FVs, no significant difference was detected between SEA Condamine control and LMA-affected affected flours for both WF and WWF. This indicates that LMA in affected WF and WWF was only sufficient to have appreciable impact on pasting development toward the end of the pasting cycle (Figure 1). Both the Chara LMA-affected WF and WWF had their RVA parameter values near the RVA detection threshold, similar to what had been reported previously [7,8,25]. The times for LMA-affected flours to reach a plateau after initial pasting occurred were almost twice as short as those of their corresponding controls. Furthermore, retrogradation, characterised by an increase of viscosity after a decline from PV, did not occur after the plateau. This indicates that LMA in the affected flour accelerated the breakdown of starch granules and hydrolysed it into small sugar fragments. Therefore, they were unable to develop a paste viscosity without enough starch for recrystallization during cooling. During gelatinization, starch granules lose their crystalline structure and become amorphous at 90 °C [26,27]. During this time of temperature transition, starch granules gradually swell to their maximum, which increases the rate of *α*-amylase hydrolysis, due to greater accessibility through the enlarged channels into the inner granules [28]. In the present study, Chara LMA-affected flours no longer pasted at 72 °C (Figure 3A). This suggests that starch granules were already extensively broken down by LMA without reaching maximum swelling, and that the amount of LMA in affected flours was adequate to hydrolyse it into small fragments and impede pasting. This is supported by the findings of Singh and Kayastha [29], in which the enzymatic activity of wheat *α*-amylase was observed to be high between 70–75 °C, and were only 20% lower than its peak at 68 °C.

Silver nitrate was used to examine LMA and residual *α*-amylase impact on LMA-affected and control flour pasting properties. Silver nitrate restored the inherent pasting properties of both cultivars, LMA-affected WF and WWF, and all viscosity-related RVA parameters were significantly higher than their corresponding controls (Table 2).

The results from this section highlight two points. First, the inherent *α*-amylase in control flours has a notable impact on pasting performance, corresponding to that observed in the FN results for Chara control flours with silver nitrate. Olaerts et al. [23] reported a similar observation, in which the inherent *α*-amylase in sound wheat flours caused significant reduction of FN values and RVA pasting viscosity parameters. Second, the higher pasting ability of LMA-affected flours in the presence of silver nitrate suggests that LMA in matured grains did not have detrimental effects on native granular starch, until thermal processing. Thus, the LMA-affected flours had better pasting properties than their controls when LMA was deactivated upon using silver nitrate. It was also shown in previous studies that there was little to no difference in the physical structure of starch granules, molecular starch structure, and other non-polysaccharide components between LMA-affected and sound wheat [4,7,10]. For SEA Condamine, silver nitrate only increased the FV of LMA-affected WF and WWF significantly, whilst other RVA parameters were similar to those of their respective controls. This indicates that the low amount of LMA or total *α*-amylase in sound grains was still able to hydrolyse the leached polymers to an extent it affected the formation of the gel network during pasting.

### 3.4. Gelation Characteristics of LMA-Affected WFs in the Presence of Oil, Salt and Milk during Thermal Processing

**Figure 4:**
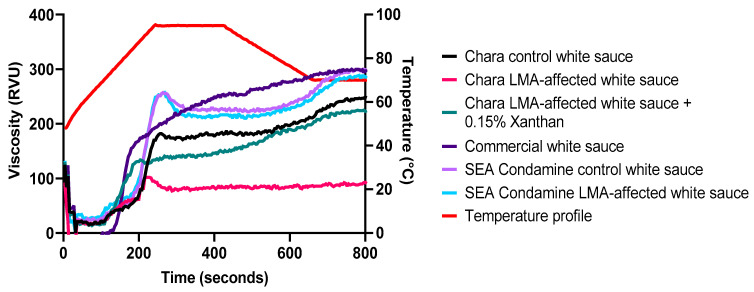
Gelation behaviour of white sauces prepared from control and LMA-affected flour during thermal processing.

An RVA was employed for making white sauce to examine the impact of LMA on semi-solid food viscosity development in the presence of other ingredients. Xanthan, a common food thickener used in sauces and dressings, was added to Chara LMA-affected white sauce to examine the gelation enhancement effect that it produces. As the difference between SEA Condamine control and LMA-affected flour for their pasting and gelation properties was not large enough for meaningful comparison, xanthan was excluded for this set of samples. In addition to Chara and SEA Condamine, gelation and texture properties of a commercial white sauce was also evaluated. It is noted that the objective of the inclusion of a commercial white sauce in the present study is not to make comparison between white sauces prepared in this work and commercial white sauce, but to relate various experimental results with well-characterized white sauces to what is seen in a commercial product. The PV and FV in gelation are defined as the highest viscosity developed during the heating stage and the viscosity at the end of the test, respectively. As shown in Figure 4, all white sauces exhibited both shear-thickening and slight shear-thinning behaviour. Similar to what was observed in the RVA, SEA Condamine control and LMA-affected white sauce have minor, if any, differences in their gelation properties. They have the same PV (257 RVU), but the former has FV (299 RVU), that is 3.3% higher than the latter. After reaching their PV, the viscosity of both white sauces gradually declined, more notably that for LMA-affected white sauce. Their difference in viscosity remained constant until the end of the test. The difference in gelation behaviour was more striking for the Chara control and LMA-affected white sauce. The RVA parameters for the former were 182 RVU PV and 250 RVU FV, which were higher than the latter by factors of 2.1 and 2.7, respectively. Evidently, a 0.5 OD LMA content has significant detrimental effects on white sauce gelation properties. However, unlike what was observed in the RVA pasting properties, the LMA-affected white sauce was able to develop viscosity and maintain its gelation when processed with other ingredients, albeit having a lower WF ratio (6%) to its total mixture than that of an RVA suspension of WF (14%) to water ratio. The addition of xanthan markedly enhanced Chara LMA-affected white sauce gelation properties, PV and FV being increased by 36.6% and 140% respectively. The gelation characteristics among Chara white sauces were distinctively different. LMA-affected white sauce with xanthan thickened gradually after its PV, whilst the control white sauce viscosity remained constant after its PV and only increased markedly towards the end of cooling and the holding temperature stage of thermal processing. In contrast, LMA-affected white sauce viscosity was consistent following a decline after reaching its PV. This indicates that Chara LMA-affected white sauce gelation development after PV was hindered by the high amount of LMA content in its sauce. On the basis that gelation is the process in which leached starch polymers aggregate, predominately amylose, during gelation, LMA may have hydrolysed the starch chains to an extent that was unfavourable for double helix formation and aggregation [30,31,32,33]. Thus, they were unable to develop a more viscous gel during the cooling stage.

These results show that oil (lipids), salt (sodium chloride), skimmed milk and xanthan, were able to develop and maintain gelation of white sauce made from high LMA WF to a marked extent. Studies have demonstrated the individual and synergistic effects of exogenous protein, lipids and salt in flour-water suspensions in facilitating viscosity development, and henceforth indirectly limiting the rate of enzymatic hydrolysis in flour–water colloidal systems [34,35,36,37]. For instance, lipids are able to interact with leached amyloses to form amylose–lipid complexes (ALC) and prevent starch granules from over-swelling. This limits enzyme accessibility into the inner granules and consequently slows down the rate of hydrolysis [35]. Furthermore, ALC can also retain the rigidity of starch granules, and this structure integrity also contributes to overall viscosity [31]. According to Ahmad and Williams [34], salt promotes amylose aggregation and the formation of the three-dimensional network gel during pasting. This effect is known as salting-out (structure-making), which strengthens the ionic interaction and hydrogen bonds between starch molecules. As a result, a more viscous gel is developed, as the leached polymers can aggregate into a denser matrix upon cooling, and this newly formed structure is not ideal for enzymes to bind onto the surface of starch, which reduces the rate of enzymatic activity [38]. Other than xanthan’s well-known thickening effect, previous studies have also reported its ability to reduce enzyme catalytic efficiency [37,39]. Although the mechanism of action is not entirely understood, it was proposed that xanthan can interact with leached polymers to form a more complex matrix around the swollen granules, making it more rigid, and this conformation is an effective barrier for resisting enzymatic activity [37]. Samutsri and Suphantharika [40] reported that rice starch paste that contained 0.3% xanthan had a higher final FV than its control. Furthermore, when 0.1 M salt solution was used in place of distilled water, it was observed that the 0.3% xanthan-starch paste had an FV 2.25 times higher than its control. The gelation development observed in LMA-affected white sauce was the outcome of combined effects of interaction among all the ingredients during thermal processing. Hence, it is hard to specify the specific contribution of individual components towards the reduction of enzymatic activity and gelation development. Nonetheless, these results show that LMA-affected flour is still functional to a considerable extent in the presence of other components, thus demonstrating its suitability for processing food products of lower viscosity.

### 3.5. Texture Properties of Fresh White Sauce

The texture parameter values of back extrusion measurement are summarized in Table 3. For SEA Condamine, all texture parameters for LMA-affected white sauce were lower than that of the control white sauce. However, only the consistency and IOV between the two white sauces were significantly different. Their parameter differences ranged between 2.94 and 5.7%. For Chara, the impact of LMA on white sauce texture properties was more pronounced, due to the high LMA content in its affected-WF. All texture parameter values of the control white sauce were 1.6 to 2.04 times higher than that of LMA-affected white sauce. Similar to the earlier observation in this study, xanthan greatly enhanced the overall texture properties of LMA-affected white sauce. There was no significant difference in firmness and cohesiveness between the Chara control and 0.15% xanthan LMA-affected white sauce. Nonetheless, the consistency and IOV of the former were 5% and 12% higher than those of the latter.

The different parameters of the back extrusion technique give an inter-related aspect of white sauce physical characteristics [41]. Their values indicate the amount of force applied at compression and extrusion during the measurement, which reflects the gel strength of white sauce. In a fresh starch-hydrocolloid composite paste, gel strength is predominately related to the availability and suitability of leached starch polymers, primarily amylose, for the formation of the semi-crystalline network upon cooling after gelatinization [42]. Thus, any discrepancy in parameter values between control and LMA-affected white sauce highlights the impact of LMA on starch recrystallization, thereby revealing its detrimental effects on white sauce [36]. The significant reduction in the consistency and IOV for both Chara and SEA Condamine LMA-affected white sauces shows that their physical qualities were compromised, dependent on the amount of LMA in WF (Figure 1 and Table 3). The lower consistency and IOV of LMA-affected white sauces indicate that there was lesser engagement between the leached starch polymers hydroxyl groups and water in the continuous phase [42]. This suggests that LMA may have resulted in hydrolysis of the leached polymers into smaller chain fragments during processing. Consequently, less water was immobilized by the intramolecular hydrogen bonds of starch molecules, resulting in the formation of a lower viscosity gel. Although firmness and cohesiveness for SEA Condamine LMA-affected white sauce were not significantly different from their controls, these parameters may not be the best indicators for assessing the overall physical quality of white sauce. This is in view of the fact that the values of firmness and cohesiveness are determined based on a single point of contact at which maximum force was applied, during the compression and extrusion respectively. In contrast, consistency and IOV parameter values were derived from the total force exerted over the entire course of back extrusion technique, thus making their values more representative of the overall physical quality of the white sauce. Consistent with the results of the gelation behaviour of LMA-affected white sauce, the presence of 0.15% xanthan greatly enhanced Chara LMA-affected white sauce texture properties, for which no parameters were radically different from those of the control white sauce. This further highlights that the detrimental effects of LMA observed in earlier evaluation can be mitigated and the loss of viscosity due to high enzymatic activity in LMA-affected flour can be compensated by other ingredients; more notably by xanthan, as demonstrated in this study, during processing. Based on evidence of earlier studies and present findings, using xanthan or incorporating other hydrocolloids in combination would potentially reinforce LMA-affected white sauce viscosity and texture properties to values similar to the commercial standard [37,40,42].

## 4. Significance of Present Study and Conclusions

The present findings are contrary to previous studies on a kindred system, which reported that LMA had no detrimental effects on bread quality [7,8]. This discrepancy is attributed to the difference in moisture content between the two foods: bread and white sauce have typical moisture contents of 50% and 90% respectively. Slaughter et al. [43] reported that the catalytic efficiency of pancreatic *α*-amylase was 5.6 times higher in 40% moisture than in 10% moisture heat processed wheat starch suspensions. It was also observed that the starch granules from a wheat starch suspension with 10% moisture were significantly less deformed than the latter, hence explaining the lower rate of hydrolysis. These observations were consistent with those of Wang and Copeland [44], which showed that the granular structure of gelatinized starch with 56% moisture content was still intact, whilst granules of gelatinized starch with 92% moisture were completely broken down into gel film, causing them to be more susceptible to enzymatic hydrolysis. As opposed to the FN and pasting properties of this study, the gelation of LMA-affected WF and their white sauce texture properties clearly demonstrates their functionality in presence of other ingredients during processing. Although a high amount of LMA in WF compromised some of the white sauce’s overall physical qualities, downgrading them to feed grade is nonetheless excessive.

Taken altogether, this study highlights the needs for alternative approaches to determine grain quality and a re-evaluation on current grain quality classification for managing LMA-affected grains, to minimise wastage, resources and economic losses.

## Figures and Tables

**Table 1:** Falling Number of control and LMA-affected flours. Results are presented as mean of biological quadruplicate ± standard deviation. FNs of the same cultivar and flour type marked by different letter indicate significant differences (*p* ≤ 0.05).

Falling Number	Chara				SEA Condamine			
	White Flour		Whole-Wheat Flour		White Flour		Whole-Wheat Flour	
Suspension(s)	Control	LMA-Affected	Control	LMA-Affected	Control	LMA-Affected	Control	LMA-Affected
Water	306 ± 17 ^a^	99 ± 19 ^b^	240 ± 16 ^a^	69 ± 2 ^b^	380 ± 3 ^ab^	374 ± 5 ^b^	371 ± 3 ^a^	360 ± 5 ^b^
Water +2 mM silver	375 ± 6 ^c^	371 ± 9 ^c^	364 ± 3 ^c^	361 ± 8 ^c^	384 ± 4 ^a^	387 ± 4 ^a^	393 ± 3 ^c^	389 ± 5 ^c^

**Table 2:** Pasting parameters of control and LMA-affected flours. Values in parenthesis are samples evaluated in water + 2 mM silver nitrate during RVA analysis. Results are presented as mean of biological quadruplicates ± standard deviation. Values marked by different letters for the same cultivar within a row indicate significant differences (*p* ≤ 0.05).

	**Chara**			
	**White Flour**		**Whole-Wheat Flour**	
**RVA Parameter(s)**	**Control**	**LMA-affected**	**Control**	**LMA-affected**
Peak viscosity (RVU)	234 ± 3 ^a^	18 ± 3 ^b^ (260 ± 3) ^c^	204 ± 2 ^a^	12 ± 3 ^b^ (226 ± 3) ^c^
Peak time (s)	328 ± 1 ^a^	171 ± 2 ^b^ (328 ± 1) ^a^	348 ± 2 ^a^	187± 1 ^b^ (348 ± 1) ^a^
Trough viscosity (RVU)	150 ± 1 ^a^	7 ± 1 ^b^ (164 ± 1) ^c^	127 ± 3 ^a^	5 ± 1 ^b^ (137 ± 3) ^c^
Final viscosity (RVU)	289 ± 5 ^a^	7 ± 1 ^b^ (318 ± 3) ^c^	235 ± 3 ^a^	5 ± 1 ^b^ (254 ± 5) ^c^
	**SEA Condamine**			
	**White Flour**		**Whole-Wheat Flour**	
**RVA Parameter(s)**	**Control**	**LMA-affected**	**Control**	**LMA-affected**
Peak viscosity (RVU)	372 ± 3 ^a^	368 ± 3 ^a^ (368 ± 2) ^a^	343 ± 4 ^a^	344 ± 2 ^a^ (343 ± 3) ^a^
Peak time (s)	351 ± 3 ^a^	347 ± 3 ^a^ (347 ± 1) ^a^	368 ± 2 ^a^	368 ± 2 ^a^ (369 ± 1) ^a^
Trough viscosity (RVU)	230 ± 3 ^ab^	227 ± 3 ^a^ (235 ± 1) ^b^	212 ± 2 ^ab^	209 ± 2 ^a^ (215 ± 2) ^b^
Final viscosity (RVU)	409 ± 3 ^a^	395 ± 6 ^b^ (421 ± 3) ^c^	370 ± 6 ^a^	359 ± 8 ^b^ (383 ± 2) ^c^

**Table 3:** Comparison of white sauce texture properties processed from control and LMA-affected white flour. Results are presented as mean of biological quadruplicates ± standard deviation for Chara and SEA Condamine, whereas results are presented as technical quadruplicates ± standard deviation for commercial white sauce. Values marked by different letters for the same cultivar within a column indicate significant differences (*p* ≤ 0.05). Commercial white sauce texture properties results were not included in the statistical comparison.

	Firmness (g)	Consistency (g s)	Cohesiveness (g)	Index of Viscosity (g s)
Chara				
Control white flour	42.7 ± 1.4 ^a^	563.0 ± 19.6 ^a^	−21.0 ± 2.5 ^a^	−224.4 ± 12.5 ^a^
LMA-affected white flour	20.9 ± 0.3 ^b^	268.6 ± 4.3 ^b^	−12.9 ± 0.3 ^b^	−132.0 ± 0.3 ^b^
LMA-affected white flour + 0.15% xanthan	40.0 ± 2.3 ^a^	534.9 ± 14.2 ^c^	−18.7 ± 1.7 ^a^	−197.0 ± 7.5 ^c^
SEA Condamine				
Control white flour	50.9 ± 4.5 ^a^	676.7 ± 11.5 ^a^	−29.1 ± 2.9 ^a^	−321.8 ± 4.8 ^a^
LMA-affected white flour	49.4 ± 3.1 ^a^	637.5 ± 18.8 ^b^	−28.0 ± 4.3 ^a^	−306.5 ± 7.5 ^b^
Commercial White Sauce	52.0 ± 0.5	685 ± 3.2	−30.8 ± 0.4	−323.8 ± 2.0
